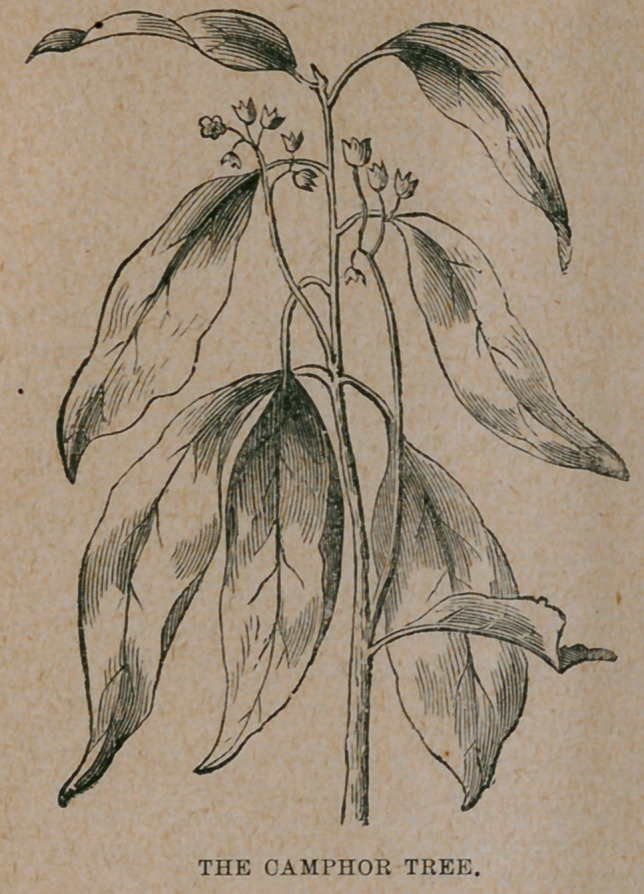# The Camphor Tree

**Published:** 1889-03

**Authors:** 


### THE CAMPHOR TREE.

The *Laurus camphora* of china is an evergreen of the laurel family, having glossy leaves and bearing clusters of yellowish flowers, which are succeeded by bunches of fruit resembling black currants. This valuable tree, which often adorns the banks of the rivers, was in several places found by Lord Amherst's embassy above 50 feet high, with its trunk 20 feet in circumference. The Chinese themselves affirm that it sometimes attains the height of more than 300 feet, and a circumference greater than the extended arms of twenty men could embrace. Camphor is obtained from the branches by steeping them, while fresh cut, in water for two or three days, and then boiling them till the gum, in the form of a white jelly, adheres to a stick which is used in constantly stirring the branches. The fluid is then poured into a glazed vessel, where it concretes in a few hours. To purify it the Chinese take a quantity of finely powdered earth, which they lay at the bottom of a copper basin ; over this they place a layer of camphor, and then another layer of earth, and so on until the vessel is nearly filled, the last or topmost layer being of earth. They cover this last layer with leaves of a plant called poho, which seems to be a species of mentha (mint). They now invert a second basin over the first, and make it air-tight by luting. The whole is then submitted to the action of a regulated fire for a certain time, and then left to cool gradually. On separating the vessels the camphor is found to have sublimed and to have adhered to the upper basin. Repetitions of the same process complete its refinement. Besides yielding this invaluable ingredient, the camphor tree is one of the principal timber trees of China, and is used not only in building, but in most articles of furniture. The wood is dry and of a light color.

THE CAMPHOR TREE.

In the Island of Sumatra there is a variety of the camphor tree which is much larger than that of China, under the bark of which the gum is found in a concrete form, and from which it is brushed down carefully with long brooms. Another variety of the same tree yields its gum in the form of pith. In this case the gathers first pierce the trees with an axe to discover their worth, as no outward sign betrays whether the heart of the tree will be found to contain oil, a resinous pitch, or gum. After the axe has disclosed the white and shining substance for which they seek, the tree is cut down, divided into lengths of about 3 feet, and split open very carefully, when the gum is taken out in solid rolls, often as large as a man's arm, and all ready for market. One tree sometimes furnishes as much as eleven pounds of gum, of so fine a quality as to be valued by the Chinese at fifty times the price of that produced by their own trees. This superior quality, of which the Island of Sumatra yields only about three hundred pounds a year, is rarely, if ever, exported.

The mode of preparing camphor for commerce as practiced by the Japanese is a species of distillation. The wood is cut up into chips and these are put into a tub fitted over a large iron pot under which a slow fire is kept. The pot is partially filled with water and the bottom of the tub is pierced with holes through which the steam from the heated water passes. The tub has a close-fitting cover, and a bamboo pipe connects it with a series of other tubs. The steam passing through the chips separates the oil and camphor from them, and in the last tub the cooled extract falls upon straw which catches the camphor crystals while the oil and water escape and are held in a lower compartment. The camphor is collected and packed for market, and the oil is used for illuminating and other purposes.

## Figures and Tables

**Figure f1:**